# Association between adjuvant radiation treatment and breast cancer‐specific mortality among older women with comorbidity burden: A comparative effectiveness analysis of SEER‐MHOS

**DOI:** 10.1002/cam4.6493

**Published:** 2023-09-14

**Authors:** Eunkyung Lee, Robert B. Hines, Jianbin Zhu, Michael J. Rovito, Kavita V. Dharmarajan, Madhu Mazumdar

**Affiliations:** ^1^ Department of Health Sciences University of Central Florida College of Health Professions and Sciences Florida Orlando USA; ^2^ Department of Population Health Sciences University of Central Florida College of Medicine Florida Orlando USA; ^3^ Department of Statistics and Data Science University of Central Florida College of Sciences Florida Orlando USA; ^4^ Research Institute, Advent Health Florida Orlando USA; ^5^ Department of Radiation Oncology, Department of Geriatrics Palliative Medicine Icahn School of Medicine at Mount Sinai New York New York USA; ^6^ Institute for Healthcare Delivery Science Icahn School of Medicine at Mount Sinai New York New York USA

**Keywords:** cancer‐specific mortality, comorbidity burden, latent class analysis, low‐risk breast cancer, radiotherapy, risk stratification

## Abstract

**Background:**

The National Comprehensive Cancer Network suggested that older women with low‐risk breast cancer (LRBC; i.e., early‐stage, node‐negative, and estrogen receptor‐positive) could omit adjuvant radiation treatment (RT) after breast‐conserving surgery (BCS) if they were treated with hormone therapy. However, the association between RT omission and breast cancer‐specific mortality among older women with comorbidity is not fully known.

**Methods:**

1105 older women (≥65 years) with LRBC in 1998–2012 were queried from the Surveillance, Epidemiology, and End Results–Medicare Health Outcomes Survey data resource and were followed up through July 2018. Latent class analysis was performed to identify comorbidity burden classes. A propensity score‐based inverse probability of treatment weighting (IPTW) was applied to Cox regression models to obtain subdistribution hazard ratios (HRs) and 95% CI for cancer‐specific mortality considering other causes of death as competing risks, overall and separately by comorbidity burden class.

**Results:**

Three comorbidity burden (low, moderate, and high) groups were identified. A total of 318 deaths (47 cancer‐related) occurred. The IPTW‐adjusted Cox regression analysis showed that RT omission was not associated with short‐term, 5‐ and 10‐year cancer‐specific death (*p* = 0.202 and *p* = 0.536, respectively), regardless of comorbidity burden. However, RT omission could increase the risk of long‐term cancer‐specific death in women with low comorbidity burden (HR = 1.98, 95% CI = 1.17, 3.33), which warrants further study.

**Conclusions:**

Omission of RT after BCS is not associated with an increased risk of cancer‐specific death and is deemed a reasonable treatment option for older women with moderate to high comorbidity burden.

## 1 INTRODUCTION

Breast cancer remains the most common cancer and the second leading cause of cancer deaths among United States (US) women,^1^ with an estimated 293,790 new cases and 43,170 deaths in 2023.^2^ The number of older women (≥65 years) with breast cancer continues to grow with aging populations^3^ and approximately 46% of the new breast cancers occurring in women aged 65 and older.^3^ Those diagnosed with early‐stage cancer have multiple options for locoregional treatment depending on many factors, including type and stage of the tumor. Since breast‐conserving therapy (i.e., breast‐conserving surgery [BCS] + adjuvant radiation treatment [RT]) demonstrated efficacy similar to mastectomy but less arm‐related symptoms and more positive perceptions of body image,^4^, ^5^ it has been one of the standard treatment options for early‐stage breast cancer, with about 50% of those patients undergoing RT after BCS in 2022.^6^

However, there has long been a debate as to whether adjuvant RT is necessary for older women considering their lowered overall life expectancy and reduced recurrence rates.^7^ For example, the Cancer and Leukemia Group B (CALGB) 9343 randomized controlled trial showed that the 5‐year recurrence rates were very low (4% without RT vs. 1% with RT, *p* < 0.001)^8^ regardless of receipt of RT after BCS, in women over 70 and diagnosed with low‐risk (i.e., early‐stage, node‐negative, and estrogen receptor [ER]‐positive) breast cancer (LRBC). However, the two groups had no significant difference in 5‐year overall survival rates (86% without RT and 87% with RT, *p* = 0.94).^8^ Based on these results, the National Comprehensive Cancer Network (NCCN) modified Breast Cancer Guideline in 2004, suggesting that RT omission is a reasonable clinical option for older women with LRBC if they were to be treated with hormone therapy.^9^ The 5‐year results of the PRIME II trial with women 65 years of age and older^10^ and 10‐year follow‐up results of CALGB 9343 and PRIME II trials further confirmed that adjuvant RT could be omitted in older women with LRBC.^11^, ^12^

There is wide variation, however, in this guideline's implementation within clinical practice. The pattern of RT omission is inconsistent across institutions, including NCCN facilities.^13^, ^14^, ^15^ More importantly, more than 50% of older women still receive radiation treatment.^16^ A survey conducted in a large cancer center in the United States reported that due to challenges in estimating the life expectancy of older women,^13^ 39% of surgeons and radiation oncologists overestimated survival benefits from additional RT.^17^ Many treatment‐decision algorithms use patient's age to assess life expectancy, but chronological age may not always correlate with one's biological age and risk of death. There have been many efforts to identify a group of women with breast cancer who may have a higher risk of mortality using a comorbidity score, such as the Charlson Comorbidity Index (CCI) or a CCI‐based index.^18^, ^19^ However, accurate calculation of these comorbidity scores requires an extensive examination of electronic medical records. Despite this, they may not capture other aspects of health that are important for predicting outcomes in older patients. Other research used latent class analysis (LCA) to capture patients' comorbidity profiles from the population‐based cancer registry databases, where extensive medical records and the severity of health conditions are unavailable.^20^, ^21^ One example is the Surveillance, Epidemiology, and End Results (SEER)‐Medicare Health Outcomes Survey (MHOS) linked data resource, where patient‐reported comorbid conditions could be used for risk stratification for treatment. We included functionalities and symptoms as well, as they may reflect the severity and burden of the comorbid conditions.

Thus, the purpose of the present study was twofold. First, we aimed to stratify older women with LRBC queried from SEER‐MHOS based on their comorbidity and symptom burden using LCA. Next, we assessed the association between RT omission and breast cancer‐specific mortality, overall and separately for each comorbidity burden class.

## 2 METHODS

### 2.1 Data source: SEER‐MHOS

This retrospective cohort study utilized SEER‐MHOS data resources. The SEER cancer registry provides highly accurate and reliable patient data regarding demographics, initial treatment, sequence of treatments, tumor characteristics, vital status, and cause of death. The SEER program, which began in 1973, currently covers nearly 48% of the US population including 20 registries.^22^ The MHOS is a longitudinal survey conducted by the Centers for Medicare & Medicaid Services to gather valid and reliable clinically meaningful data, including patient‐reported comorbidity, functionality, and symptoms, among a random sample of Medicare Advantage beneficiaries. The MHOS started in 1998 and is conducted annually. Therefore, the SEER‐MHOS linked data resource provides researchers an opportunity to examine patient‐reported outcomes among Medicare Advantage beneficiaries with cancer diagnosis.

### 2.2 Study population

The study protocol received institutional review board approval and has been reported in detail previously.^23^ We selected older women with LRBC to ensure BCS and hormone therapy was the primary treatment, and RT could be an option. The inclusion criteria included: (1) a diagnosis of primary breast cancer between 1998 and 2012 (to ensure at least 5 years of follow‐up after cancer diagnosis) and reported to SEER, (2) a diagnosis of LRBC (i.e., tumor stage = “0, I, or II,” number of positive node = “0,” and ER status = “positive”), (3) ≥65 years old at diagnosis, (4) receipt of BCS as the primary treatment for breast cancer, and (5) completion of at least one survey within 24 months before cancer diagnosis. Participants were excluded from the analysis if they: (1) had a previous cancer diagnosis (other than non‐melanoma skin cancer), (2) had more than one primary tumor at diagnosis, (3) received treatment outside of standard time frames (i.e., treatment initiated 1 year after cancer diagnosis), (4) received RT before surgery, or (5) had missing information on the vital status.

As individuals provided informed consent to participate in future research during the MHOS survey, this study is exempt from obtaining informed consent from study participants.^24^

### 2.3 Measurements

#### 2.3.1 Sociodemographic and tumor‐related factors

Sociodemographic (race/ethnicity, marital status, education, smoking status, household income, and health insurance coverage) factors were obtained from the enrollment database maintained by the Centers for Medicare & Medicaid Services and/or self‐reported information in the MHOS survey. Tumor‐related factors (age at diagnosis, diagnosis year, disease stage, grade, hormone receptor status, and geographic regions) were obtained from the SEER‐Patient Entitlement and Diagnosis Summary File (PEDSF). The information regarding human epidermal growth factor receptor 2 (HER2) status is not included in the current analysis, as HER2 testing was unavailable until 2010.

#### 2.3.2 Radiation treatment

Treatment status was defined as BCS + RT or BCS alone according to information on the type of surgery, RT, and the sequence of these two variables in the SEER data file. Lumpectomy, segmental resection, quadrantectomy, or partial mastectomy are examples of BCS. Based on the sequence of surgery and RT, only RT received after surgery was considered in the RT group. Data on the first course treatment (i.e., surgery and RT) in the SEER data file are considered generally reliable^24^ with a high positive predictive value of 95% for RT.^25^

#### 2.3.3 Comorbidity, functionality, and symptoms

The MHOS questionnaire includes questions about the presence of 12 comorbid conditions (angina pectoris/coronary artery disease, hypertension, acute myocardial infarction, congestive heart failure, stroke, chronic lung disease, diabetes, inflammatory bowel diseases, arthritis, heart attack, sciatica, and depression), which were assessed by the question, “has a medical doctor ever told you that you had [condition]?” They were coded dichotomously (1 = “yes,” 2 = “no”). There is no reliability reported for comorbidity data in MHOS. However, the reliability of self‐reported comorbidities similar to ones in MHOS was moderate with varying values depending on the type of conditions.^26^ For example, the kappa value was 0.14 for arthritis and 0.76 for diabetes among older adults.^26^

The limitation of functionality was assessed with the following seven questions: six questions on activities of daily living (ADLs; bathing, dressing, eating, using the toilet, walking, and getting in or out of chairs) and one question on moderate activities, with each activity being considered individually in the LCA. They were coded dichotomously (1 = “I am unable to do this activity” or “yes, I have difficulty,” 2 = “no”).

Symptoms were assessed by the two questions about chest pain (on exercise or when resting) and the three questions about shortness of breath (when resting, walking, and climbing stairs), and they were coded dichotomously (1 = “all of the time,” “most of the time,” or “some of the time,” 2 = “no”). We used information from surveys completed within 24 months before cancer diagnosis to assess the pre‐diagnosis comorbidity burden. If participants completed more than once, the most recent one in relation to the diagnosis date was used.

#### 2.3.4 Cancer‐specific deaths

The primary outcome was survival time, defined as the time from the date of diagnosis to the date of death or censoring at the last follow‐up, July 30, 2018, whichever came first. The date and causes of death were verified through the National Death Index.

### 2.4 Statistical analysis

Baseline characteristics of the study population were described using mean and standard deviation (SD) or frequency and percentage and compared by treatment status (BCS + RT vs. BCS only) using *t*‐tests or chi‐squared tests appropriately.

#### 2.4.1 Latent class analysis

Since a very small number of events were observed in the current study (i.e., only 47 cancer‐specific deaths), not all covariates could be included individually in the multivariable survival analysis because one variable per 10 events is a widely accepted minimal criterion for adequate parameter estimation.^27^ To alleviate this issue, we examined latent comorbidity and symptom burden classes with the aforementioned 24 observed items and included only the latent class membership in the subsequent multivariable analysis. Using the following steps, the LCA modeling was performed in Mplus version 8.6 (Muthén & Muthén).^28^ First, preliminary latent class models with increasing numbers of classes (1–6 classes) without covariates were fit to the data. Second, the optimal model was identified by comparing model fit statistics, such as the Akaike information criterion (AIC), the Bayes information criterion (BIC), sample size‐adjusted BIC (SABIC), Vuong‐Lo–Mendell–Rubin (VLMR) likelihood ratio tests (LRTs), and entropy as well as by considering the clinical interpretation of comorbidity burden and statistical power in the subsequent analyses. The lowest values of AIC and BIC indicate the best‐fitting model given the data. A non‐significant *p*‐value (≥0.05) from LRT suggests that a model with k‐1 classes is sufficient and that k classes are not better than k‐1 classes. When consensus is not reached, guidance from LRT is used. Third, the item‐response probability for each item was examined to describe the characteristics of each class. Finally, individuals were allocated to their most probable class based on the posterior class probabilities. When missingness is found in data, Mplus estimates the latent class membership assuming missing at random, and the full information maximum likelihood is utilized.

#### 2.4.2 Unadjusted analysis for overall survival and cumulative incidence of cancer‐specific deaths

The Cox regression model was used to obtain unadjusted estimates for 5‐, 10‐, and 20‐year overall survival with 95% confidence intervals (CIs). The modified Cox model was used to obtain unadjusted estimates of 5‐, 10‐, and 20‐year cumulative incidence of cancer‐specific deaths according to RT omission and comorbidity burden. The difference in cumulative incidence function according to RT omission was tested using Gray's tests.

#### 2.4.3 Inverse probability of treatment weighting‐adjusted survival analysis

In this observational comparative effectiveness study, where treatment was not randomized, potential bias due to treatment selection and confounding could impact the estimate of the treatment effect.^29^ Therefore, we used the inverse probability of treatment weighting (IPTW) method.^30^, ^31^ The propensity score, the probability of receiving the treatment (i.e., RT), for an individual was estimated in generalized boosted models using the R package, twang, with receiving RT as the dependent variable and the baseline sociodemographic and tumor‐related factors listed above as independent variables. Then, the inverse of this probability was included in subsequent analyses as a weight.^32^ Differences in potential confounding variables between the two treatment groups were evaluated by the standardized mean differences.^33^, ^34^

The Cox proportional hazards model was then used to obtain IPTW‐adjusted subdistribution hazard ratios (HRs) and 95% CIs for 5‐, 10‐, and 20‐year cancer‐specific mortality due to RT omission considering non‐cancer‐related deaths as a competing risk, overall and separately by comorbidity burden class. All analyses were conducted using SAS 9.4, and statistical significance was set with *p* < 0.05.

## 3 RESULTS

### 3.1 Study participants

**FIGURE 1:**
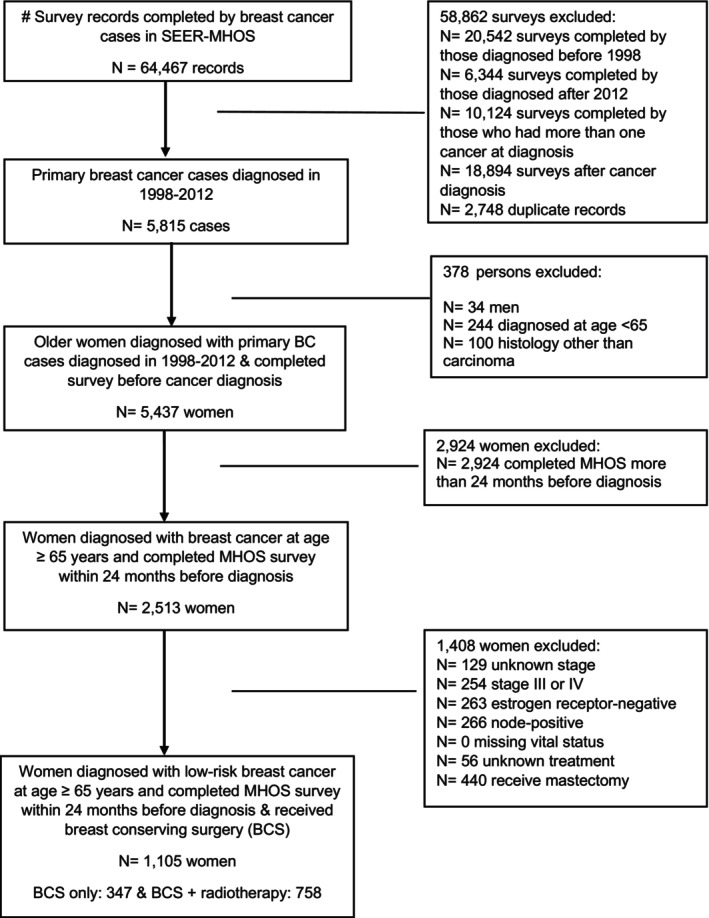
Flow diagram of cohort selection.

Among 64,467 survey records in the SEER‐MHOS breast cancer data file, 5437 older women diagnosed with primary breast cancer in 1998–2012 and completed surveys before diagnosis were identified. Women should meet all three conditions to be eligible. (1) diagnosis with LRBC, (2) received BCS, and (3) completed surveys within 24 months before cancer diagnosis were queried. The average (± standard deviation) time between survey completion and cancer diagnosis was 11.4 ± 6.7 months before cancer diagnosis. The detailed selection process is described in Figure 1. The sample consists of non‐Hispanic whites (74.6%), blacks/African Americans (9.3%), Hispanics (7.3%), Asian/Pacific Islanders (7.1%), and other (1.7%), and the mean age was 75.0 ± 5.9 years old. As shown in Table 1, more than two‐thirds of women (68.6%) received RT after BCS. Participants who omitted RT were more likely than those who received RT to be 75 years or older (62.8% vs. 43.4%) and unmarried (59.9% vs. 52.2%), to have a household income lower than $40,000 (55.6% vs. 59.3%), to live in the Northeast region (19.3% vs. 13.1%), and to have stage 0 disease (42.4% vs. 20.4%), tumor grade of unknown (16.7% vs. 9.2%), progesterone receptor‐negative tumor (32.3% vs. 13.1%), and comorbidities ≥3 (50.1% vs. 41.0%).

**TABLE 1:** Demographic and clinical characteristics of older women with low‐risk breast cancer, SEER‐MHOS (*n* = 1105).

Characteristics	Categories	All		BCS only		BCS + RT		*p* value
		*N*	%	*N*	%	*N*	%	
Study population		1105	100	347	31.4	758	68.6	
Median follow‐up time (years) (95% CI)		8.3 (7.9, 8.6)		7.3 (7.1, 7.9)		8.7 (8.3, 9.0)		**<0.0001**
Age at diagnosis (year)	65–69	224	20.3	42	12.1	182	24.0	**<0.0001**
	70–74	334	30.2	87	25.1	247	32.6	
	75–79	286	25.9	84	24.2	202	26.6	
	≥80	261	23.6	134	38.6	127	16.8	
Race/ethnicity	NHW	824	74.6	254	73.2	570	75.2	0.911
	NHB	103	9.3	34	9.8	69	9.1	
	Hispanic	81	7.3	29	8.4	52	6.9	
	Asian/PI	78	7.1	–		–		
	Other	19	1.7	–		–		
Marital status	Married	501	45.3	139	40.1	362	47.8	**0.009**
	Widowed	383	34.7	147	42.4	236	31.1	
	Divorced/separated	156	14.1	45	13.0	111	14.6	
	Single	33	3.0	–		–		
	Unknown	32	2.9	–		–		
Education	Less than high school	245	22.2	88	25.4	157	20.7	0.127
	High school/some college	672	60.8	206	59.4	466	61.5	
	College graduate	164	14.8	–		–		
	Unknown	24	2.2	–		–		
Smoking status	Yes	86	7.8	26	7.5	60	7.9	0.971
	No	825	74.7	260	74.9	565	74.5	
	Unknown	194	17.6	61	17.6	133	17.5	
Household income	<$20,000	344	31.1	107	30.8	237	31.3	**0.003**
	$20,000–39,999	298	27.0	86	24.8	212	28.0	
	$40,000–79,999	169	15.3	39	11.2	130	17.2	
	≥$80,000	47	4.3	15	4.3	32	4.2	
	Unknown	247	22.4	100	28.8	147	19.4	
Year of diagnosis	1998–2004	314	28.4	85	24.5	229	30.2	0.147
	2005–2007	244	22.1	80	23.1	164	21.6	
	2008–2012	547	49.5	182	52.4	365	48.2	
State buy‐in coverage	No	996	90.1	310	89.3	686	90.5	0.547
	Yes	109	9.9	37	10.7	72	9.5	
Census tract poverty level	0% to <5%	306	27.7	105	30.3	201	26.5	0.325
	5% to <10%	308	27.9	98	28.2	210	27.7	
	10% to <20%	287	26.0	83	23.9	204	26.9	
	20% to 100%	192	17.4	–		–		
	Unknown	12	1.1	–		–		
SEER region	West	587	53.1	181	52.2	406	53.6	**0.001**
	Northeast	166	15.0	67	19.3	99	13.1	
	Midwest	86	7.8	13	3.7	73	9.6	
	South	266	24.1	86	24.8	180	23.7	
Geographic residency	Big metro	655	59.3	213	61.4	442	58.3	0.620
	Metro	388	35.1	115	33.1	273	36.0	
	Other	62	5.6	19	5.5	43	5.7	
Tumor stage	Stage 0	302	27.3	147	42.4	155	20.4	**<0.0001**
	Stage I	694	62.8	168	48.4	526	69.4	
	Stage II	109	9.9	32	9.2	77	10.2	
Tumor grade	Well‐differentiated	327	29.6	99	28.5	228	30.1	**0.002**
	Moderately differentiated	454	41.1	125	36.0	329	43.4	
	Poorly /undifferentiated	196	17.7	65	18.7	131	17.3	
	Unknown	128	11.6	58	16.7	70	9.2	
Hormone receptor status	Positive	894	80.9	235	67.7	659	86.9	**<0.0001**
	Negative	211	19.1	112	32.3	99	13.1	
Number of comorbidities	0–1	345	31.2	104	30.0	241	31.8	**0.013**
	2	275	24.9	69	19.9	206	27.2	
	3	220	19.9	84	24.2	136	17.9	
	≥4	265	24.0	90	25.9	175	23.1	

*Note*: Significant findings are in bold. Some categories of data are suppressed due to individual cells *N* < 11, per the data use agreement of SEER‐MHOS.

Abbreviations: BCS, breast‐conserving surgery; NHW, non‐Hispanic White; NHB, non‐Hispanic Black; PI, Pacific Islander; RT, Radiation therapy.

### 3.2 Comorbidity burden class

The fit statistics for class enumeration in Table 2 suggested the model with three classes would fit the data best considering a non‐significant result from VLMR‐LRT comparing the model with four classes versus the model with three classes (*p* = 0.328). This observation is supported by the lowest BIC and high entropy. Although corresponding AIC and SABIC were not the lowest, their values were on the lower side. Item‐response rates are reported in Table 3 and Figure S1. Overall, 68.5% of participants reported having hypertension, 45.4% arthritis, 24.9% sciatica, and 28% depression. Regarding functional difficulties and symptoms, 58.4% had difficulties in moderate activities, and 45.7% reported shortness of breath with stairs. As shown in Figure S1, item‐response rates of comorbidity burden class indicators showed that Classes 3, 1, and 2 had relatively low, medium, and high endorsing rates for comorbid conditions, functional impairment, and symptoms, respectively. Therefore, they were named as “Low,” “Moderate,” and “High” comorbidity burden classes. “Low,” “Moderate,” and “High” comorbidity burden classes represented 55%, 36%, and 9% of the study participants, respectively. As shown in Table 4, participants in the “High” comorbidity burden class were more likely than those in the “Low” category to be 75 years and older (52.5% vs. 44.8%) and Black/African American or Hispanic (32.3% vs. 12.6%), to have state buy‐in insurance coverage (21.2% vs. 6.2%) and advanced disease with stage II (21.2% vs. 7.5%), an income lower than $20,000 (39.4% vs. 25.9%), and education less than high school (39.4% vs. 15.3%). There was a significant difference in RT omission rates across comorbidity burden classes, showing a higher omission rate in the “High” comorbidity burden class (44.4% vs. 32.7% and 28.5%, *p* = 0.005).

**TABLE 2:** Fit statistics for class enumeration for comorbidity burden.

K	AIC	BIC	SABIC	VLMR‐LRT *p*‐value	Entropy
1	22,524.4	22,644.5	22,568.3	–	–
2	19,916.6	20,162.0	20,006.4	<0.001	0.867
3	19,475.1	19,845.7	19,610.6	0.034	0.846
4	19,158.2	**19,654.0**	19,339.5	**0.328**	0.854
5	19,015.9	19,636.9	19,243.0	0.152	0.820
6	**18,903.5**	19,649.7	**19,176.4**	0.756	0.841

*Note*: Bold suggests best fit for each individual statistic.

Abbreviations: AIC, Akaike information criterion; BIC, Bayesian information criterion; K, number of classes; SABIC, sample size adjusted BIC; VLMR‐LRT, Vuong‐Lo–Mendell–Rubin‐adjusted likelihood ratio test.

**TABLE 3:** Item‐response probability of comorbidity burden indicators conditional on latent class membership.

	Comorbidity burden class			
	All	Low	Moderate	High
Comorbid conditions^a^	Endorsement: Yes (%)			
Coronary artery diseases	8.5	2.3	15.2	22.5
Hypertension	68.5	62.9	78.1	73.2
Acute myocardial infarction	5.0	2.1	9.2	8.7
Congestive heart failure	4.4	0.5	8.6	13.8
Stroke	6.5	3.8	7.6	16.7
Chronic lung diseases	14.1	6.7	25.1	23.9
Diabetes	19.1	12.0	26.7	35.5
Inflammatory bowel diseases	5.6	2.6	6.0	18.8
Arthritis	45.4	34.4	56.5	72.5
Heart attack	20.8	14.9	27.9	32.6
Sciatica	24.9	19.9	27.6	42.0
Depression	28.5	16.3	39.7	60.9
Functional difficulties^b^				
Moderate activities	58.4	40.0	79.7	96.4
Bathing	9.0	0.9	1.6	63.8
Dressing	7.5	0.0	1.6	56.5
Eating	3.4	0.5	1.0	23.2
Using the toilet	6.1	0.6	0.3	44.9
Walking	29.3	9.8	40.6	95.7
In/out of chairs	21.2	4.6	24.8	91.3
Symptoms^c^				
Chest pain with exertion	19.5	5.2	34.9	52.2
Chest pain at rest	13.6	3.4	21.3	44.2
Shortness of breath at rest	13.8	1.2	23.5	50.7
Shortness of breath walking	31.7	1.4	80.0	64.5
Shortness of breath with stairs	45.7	16.4	94.6	72.5

^a^ Chronic medical condition includes the response “Yes.”

^b^ Functional difficulties and symptoms include the responses “I am unable to do this activity” or “Yes, I have difficulty.”

^c^ Symptoms include the responses “All of the time,” “Most of the time,” and “Some of the time.

**TABLE 4:** Demographic and clinical characteristics of breast cancer patients by comorbidity burden class (*n* = 1105).

Characteristics	Categories	Comorbidity burden class						*p* value
		Low		Moderate		High		
		*N*	%	*N*	%	*N*	%	
Study population		614	55.5	392	35.5	99	9.0	
Median follow‐up time (years) (95% CI)		8.8 (8.5–9.3)		7.5 (7.3–8.2), 7.9)		6.6 (6.1–7.3)		**<0.0001**
Age at diagnosis (year)	65–69	131	21.3	67	17.1	26	26.3	**0.003**
	70–74	208	33.9	105	26.8	21	21.2	
	75–79	152	24.8	110	28.1	24	24.2	
	≥80	123	20	110	28.1	28	28.3	
Race/ethnicity	NHW	483	78.7	281	71.7	60	60.6	**<0.001**
	NHB	41	6.7	44	11.2	18	18.2	
	Hispanic	36	5.9	31	7.9	14	14.1	
	Asian/PI	43	7.0	–		–		
	Other	11	1.8	–		–		
Marital status	Married	296	48.2	168	42.9	37	37.4	0.130
	Widowed	192	31.3	153	39	38	38.4	
	Divorced/separated	87	14.2	54	13.8	15	15.2	
	Single	22	3.6	–		–		
	Unknown	17	2.8	–		–		
Education	Less than high school	94	15.3	112	28.6	39	39.4	**<0.0001**
	High school/some college	405	66.0	218	55.6	49	49.5	
	College graduate	103	16.8	–		–		
	Unknown	12	2.0	–		–		
Smoking status	Yes	37	6.0	–		–		0.184
	No	465	75.7	287	73.2	73	73.7	
	Unknown	112	18.2	–		–		
Household income	<$20,000	159	25.9	146	37.2	39	39.4	**<0.001**
	$20,000–39,999	171	27.9	101	25.8	26	26.3	
	$40,000–79,999	116	18.9	–		–		
	≥$80,000	32	5.2	–		–		
	Unknown	136	22.1	88	22.4	23	23.2	
Year of diagnosis	1998–2004	172	28.0	110	28.1	32	32.3	0.922
	2005–2007	138	22.5	85	21.7	21	21.2	
	2008–2012	304	49.5	197	50.3	46	46.5	
State buy‐in coverage	No	576	93.8	342	87.2	78	78.8	**<0.0001**
	Yes	38	6.2	50	12.8	21	21.2	
Census tract poverty level	0% to <5%	182	29.6	99	25.3	25	25.3	0.328
	5% to <10%	172	28	116	29.6	20	20.2	
	10% to <20%	149	24.3	107	27.3	31	31.3	
	20%+/Unknown	111	18.1	70	17.8	23	23.2	
SEER region	West	320	52.1	212	54.1	55	55.6	0.125
	Northeast	98	16.0	–		–		
	Midwest	58	9.4	–		–		
	South	138	22.5	98	25	30	30.3	
Geographic residency	Big metro	361	58.8	231	58.9	63	63.6	0.706
	Metro/Other	253	41.2	161	41.1	36	36.4	
Tumor stage	Stage 0	175	28.5	109	27.8	18	18.2	**<0.001**
	Stage I	393	64.0	241	61.5	60	60.6	
	Stage II	46	7.5	42	10.7	21	21.2	
Tumor grade	Well‐differentiated	180	29.3	114	29.1	33	33.3	0.760
	Moderately differentiated	256	41.7	163	41.6	35	35.4	
	Poorly/undifferentiated	104	16.9	75	19.1	17	17.2	
	Unknown	74	12.1	40	10.2	14	14.1	
Hormone receptor status	PR positive	503	81.9	307	78.3	84	84.8	0.211
	Borderline/unknown	111	18.1	85	21.7	15	15.2	
Number of comorbidities	0–1	282	45.9	–		–		**<0.0001**
	2	189	30.8	–		–		
	3	103	16.8	106	27.0	11	11.1	
	≥4	40	6.5	156	39.8	69	69.7	
Treatment	BCS only	175	28.5	128	32.7	44	44.4	**0.005**
	BCS + RT	439	71.5	264	67.3	55	55.6	

*Note*: Some categories of data are suppressed due to individual cells *N* < 11, per the data use agreement of SEER‐MHOS. Bold values indicates of the *p* value of < 0.05.

Abbreviations: BCS, breast‐conserving surgery; NHW, non‐Hispanic White; NHB, non‐Hispanic Black; PI, Pacific Islander; RT, Radiation therapy.

### 3.3 Cumulative incidence of deaths

The median follow‐up time was 8.3 years, with a significantly longer follow‐up for those with RT (RT: 8.7 years vs. no‐RT: 7.3 years) and those with low comorbidity burden (Low: 8.8 years; Moderate: 7.5 years; and High: 6.6 years) compared to their counterparts (both *p* < 0.0001). A total of 117 deaths (25 cancer‐related) occurred over the 5‐year follow‐up period, 236 women deceased (41 cancer‐related) over a 10‐year follow‐up period, and 318 deaths (47 cancer‐related) over the whole study period. In unadjusted analyses, as shown in Table 5, those who omitted RT and those in the high comorbidity burden class had significantly lower overall survival than their respective reference group. However, there was no significant difference in cumulative incidence rates of breast cancer‐specific deaths by treatment or comorbidity burden class.

**TABLE 5:** Unadjusted estimates of overall survival and the cumulative incidence of breast cancer‐specific mortality by treatment and by comorbidity burden among older women with low‐risk breast cancer, SEER‐MHOS.

	Overall survival		Cumulative incidence, breast cancer‐specific death	
	Estimate (95% CI)	*p* value	Estimate (95% CI)	*p* value
5‐year outcomes				
Treatment				
BCS + RT (*n* = 758)	91.3% (89.0, 93.1)	**0.002**	2.6% (1.6, 3.9)	0.482
BCS only (*n* = 347)	85.3% (81.1, 88.6)		1.8% (0.8, 3.9)	
Comorbidity burden				
Low (*n* = 614)	93.7% (91.4, 95.3)	**<0.0001**	1.5% (0.8, 2.8)	0.079
Moderate (*n* = 392)	86.5% (82.7, 89.5)		3.7% (2.1, 6.0)	
High (*n* = 99)	74.8% (65.0, 82.2)		2.5% (0.5, 7.7)	
10‐year outcomes				
Treatment				
BCS + RT (*n* = 758)	81.7% (74.4, 81.2)	**<0.0001**	4.6% (3.2, 6.4)	0.477
BCS only (*n* = 347)	72.1% (60.1, 71.8)		4.4% (2.1, 8.0)	
Comorbidity burden				
Low (*n* = 614)	86.2% (79.5, 86.3)	**<0.0001**	3.6% (2.2, 5.5)	0.137
Moderate (*n* = 392)	72.7% (61.1, 72.1)		5.4% (3.2, 8.3)	
High (*n* = 99)	55.6% (35.7, 58.9)		7.8% (2.7, 16.3)	
20‐year outcomes				
Treatment				
BCS + RT (*n* = 758)	75.3% (54.6, 65.3)	**<0.0001**	5.0% (3.4, 6.9)	0.894
BCS only (*n* = 347)	62.3% (25.9, 44.6)		7.6% (3.8, 13.1)	
Comorbidity burden				
Low (*n* = 614)	78.7% (55.1, 67.1)	**<0.0001**	5.9% (3.7, 8.9)	0.711
Moderate (*n* = 392)	64.5% (34.3, 50.4)		4.9% (3.0, 7.5)	
High (*n* = 99)	51.5% (24.0, 49.7)		5.7% (2.1, 12.2)	

*Note*: Significant findings are in bold.

Abbreviations: BCS, breast‐conserving surgery; CI, confidence interval; RT, Radiation therapy.

### 3.4 The association between RT omission and cancer‐specific mortality

Figure S2 shows no significant differences in potential confounding variables after IPTW adjustment between the two treatment groups. Table 6 shows that RT omission, overall, was not associated with the increased risk for cancer‐specific deaths (5‐year: HR = 0.68, 95% CI = 0.37, 1.23; 10‐year: HR = 0.87, 95% CI = 0.55, 1.36; 20‐year: HR = 1.22, 95% CI = 0.81, 1.82). In the stratified analysis according to comorbidity burden class, RT omission was not associated with the increased risk for short‐term, 5‐ and 10‐year, cancer‐specific deaths regardless of their comorbidity burden. However, among older women with low comorbidity burden, the risk for long‐term cancer‐specific deaths was significantly higher with RT omission (HR = 1.98, 95% CI = 1.17, 3.33).

**TABLE 6:** IPTW‐adjusted subdistribution hazard ratios for the association between treatment and cancer‐specific mortality, overall and by comorbidity burden class among older women with low‐risk breast cancer, SEER‐MHOS.

	5‐year outcomes			10‐year outcomes		20‐year outcomes			
	HR (95% CI)		*p* value	HR (95% CI)		*p* value	HR (95% CI)		*p* value
Treatment									
BCS + RT (*n* = 758)	Ref			Ref			Ref		
BCS only (*n* = 347)	0.68 (0.37, 1.23)		0.202	0.87 (0.55, 1.36)		0.536	1.22 (0.81, 1.82)		0.339
Low comorbidity burden									
BCS + RT (*n* = 439)	Ref			Ref			Ref		
BCS Only (*n* = 175)	0.98 (0.40, 2.40)		0.965	1.31 (0.71, 2.42)		0.382	**1.98 (1.17, 3.33)**		**0.011**
Moderate comorbidity burden									
BCS + RT (*n* = 264)	Ref			Ref			Ref		
BCS Only (*n* = 128)	0.65 (0.23, 1.52)		0.324	0.61 (0.28, 1.31)		0.205	0.61 (0.28, 1.31)		0.205
High comorbidity burden									
BCS + RT (*n* = 55)	Ref			Ref			Ref		
BCS Only (*n* = 44)	NE	NE		0.29 (0.05, 1.73)		0.175	0.29 (0.05, 1.73)		0.175

*Note*: Significant findings are in bold. IPTW model was built with age at diagnosis, race/ethnicity, marital status, year of diagnosis, insurance (state buy‐in), household income, census tract poverty level, marital status, education, urban/rural designation, SEER region, disease stage, tumor grade, and PR‐status.

Abbreviations: BCS, breast‐conserving surgery; CI, confidence interval; HR, hazard ratio; IPTW, inverse probability of treatment weighting; NE, not estimable because no death was observed in the BCS Only group; RT, radiation therapy.

The associations between RT omission and all‐cause mortality and non‐cancer‐specific mortality are displayed in Tables S1 and S2, respectively. Omission of RT increased the risk for all‐cause and non‐cancer‐specific mortality, particularly for those with moderate or high comorbidity burden. For those with low comorbidity burden, RT omission did not increase the risk for short‐term (5‐and 10‐year) all‐cause and non‐cancer specific mortality. However, the risk for long‐term all‐cause and non‐breast cancer mortality was increased with RT omission in older women with LRBC and low comorbidity burden.

## 4 DISCUSSION

This population‐based, retrospective cohort study of older women with LRBC identified three latent comorbidity and symptom burden classes. In addition, the omission of adjuvant RT after BCS was not associated with the increased risk for 5‐ and 10‐year cancer‐specific death, regardless of comorbid burden. Our results suggested a potentially increased risk for long‐term mortality with RT omission only in women with low comorbidity burden, which needs confirmation in a future randomized controlled trial. This study adds valuable empirical evidence concerning the effect of RT omission on cancer‐specific mortality utilizing patient‐reported outcomes data for risk stratification.

First, we identified three comorbidity burden classes among older women with LRBC, which were distinguished by the number and the type of comorbid conditions, the presence of functional limitation, and the severity of symptoms. About 45% of older women with LRBC had moderate or high comorbidity burden who could omit RT without scarifying breast cancer‐specific mortality. Notably, Black/African American and Hispanic women are more likely to be grouped in moderate or high comorbidity burden class in our current study and other reports,^35^, ^36^ as well as they report lower quality of life before RT initiation than their racial/ethnic counterparts.^35^ As these racial/ethnic minority groups also reported more side effects from radiotherapy,^37^, ^38^ RT omission could improve quality of life and lessen racial/ethnic disparity.

Pre‐diagnosis comorbidities in cancer patients can affect treatment decisions and prognosis.^18^, ^19^, ^39^, ^40^, ^41^, ^42^, ^43^ For example, women with breast cancer and uncontrolled diabetes mellitus were less likely to receive curative treatment than those without,^43^ which also affects prognosis. Severe comorbidity burden was associated with significantly higher all‐cause mortality.^19^ Among women with breast cancer, having diabetes mellitus was associated with an increased risk for all‐cause mortality,^43^ having a history of stroke was associated with increased non‐breast cancer mortality,^41^ while having a history of diabetes mellitus and previous cancer or myocardial infarction increased the risk for dying from breast cancer.^42^ Some studies examined the individual comorbid conditions separately,^40^, ^41^ while others examined them as a composite value using an unweighted total number^20^ or a weighted index score.^18^, ^19^, ^42^ Another approach would include comprehensive geriatric assessments (CGAs) in the treatment‐decision algorithm. One institution reduced RT usage from 63% to 29% by integrating CGAs in risk stratification^13^, ^44^ among older women eligible for RT omission based on NCCN criteria. However, CGAs are not routinely conducted in the clinic because it is time‐consuming and requires special skills by health professionals. We used LCA to identify women with varying comorbidity burdens, suggesting the potential value of patient‐reported outcomes data in risk stratification for RT omission.

About 69% of older women with LRBC received RT after BCS, which is similar to the report of 70% from a meta‐analysis of 17 trials published in 2011.^45^ It is possible that some patients were misclassified for treatment received, as RT usage for breast cancer was underreported in the cancer registries.^46^ Our observation that an RT omission rate was higher for those with high comorbidity burden reflects current clinical practice where RT tends to be omitted in those with severe comorbidity,^47^, ^48^, ^49^ as these patients have a greater risk of dying from comorbid conditions rather than from breast cancer. However, it was surprising that more than 50% of women in the “High” comorbidity burden class received RT. Although this value may not reflect the reduction of RT usage in older women in recent years,^14^ more research is warranted for older women with severe comorbidity burden to guide better‐informed and cost‐effective decision‐making for RT omission for this group of older women.

There is still substantial debate regarding subgroups of older women who can be safely treated without adjuvant RT concerning cancer‐specific survival or recurrence.^50^, ^51^, ^52^ The results from the current analysis can improve treatment decision as this study adds to evidence that RT omission could be a reasonable option for older women with LRBC, particularly those who reported moderate to high comorbidity burden, which is in line with previous prospective randomized trials, reporting no differences in overall and breast cancer‐specific survival with and without RT after BCS.^8^, ^11^, ^53^ Omission of RT can improve quality of life and reduce economic burden in both patients and healthcare system. Our findings can also inform that older women with low comorbidity burden and an anticipated extended survival period (10+ years) may benefit from RT. Considering life expectancy based on comorbidity burden levels will improve treatment decision and patient's prognosis. Evaluation of changes in quality of life according to cancer treatment in this population will provide additional evidence, which will help further with treatment decision. More evidence will facilitate implementation of this practice. In addition, the importance of risk stratification based on life expectancy and comorbidity burden should be extended to younger patients. As more evidence from clinical trials (e.g., the DEBRA‐NRG BR007 trial^54^) accumulates, RT omission could also be an option for women aged 50–70 years with LRBC.

Hormone therapy, which patients would be prescribed if RT were omitted, is not easy to tolerate.^55^ In addition, there are tremendous compliance issues,^56^, ^57^ complicating the conclusion that RT omission may be justified in patients with moderate or high comorbidity burden. In the United Kingdom (UK), a 1‐week Fast‐Forward RT regimen (26 or 27 Gy in 5 fractions over 1 week) was tested in 2011–2014 with 4096 adult women (18+ years old) with early‐stage breast cancer at 97 hospitals. The results at 5 years after randomization showed that the efficacy of a 1‐week Fast‐Forward RT regimen was not different from the current standard regimen, 40 Gy in 15 fractions over 3 weeks, in terms of ipsilateral local tumor relapse (2.1% vs. 1.4%) and patients and health professionals' evaluation of side effects. If the same results were reported for older women, a shorter RT regimen could be considered when lower compliance with hormone therapy is expected or if patients prefer to avoid the side effects of hormone therapy. Therefore, the radiation treatment paradigm may require a re‐review, given (1) the recent efficacy and safety of a 1‐week RT regimen in the United Kingdom, (2) low adherence to hormone therapy in women with breast cancer, and (3) high 15‐year survival rates, and more research, particularly in older women with comorbidity, is needed to help patients and health providers streamline treatment options leading to better survival and quality of life. When patients participated in their treatment decision‐making process, they were satisfied with clinical outcomes^58^ and reported better quality of life.^59^

There are several limitations to be noted. First, although the SEER‐MHOS data resource provides an exceptional opportunity to address cancer survivors' health outcomes, numerous innate limitations exist.^60^ For example, several prognostic factors, such as systematic therapy (e.g., chemotherapy and hormone therapy) and recurrence, which can potentially influence patient survival,^61^ are not available. We assumed that all cases were treated with hormone therapy. Also, adherence to those therapies was not available. Second, due to the retrospective nature of the study design, the possibility of selection and information bias should be considered when interpreting the results. However, these biases would likely be non‐differential and, thus, reported estimates would be biased toward the null. Third, comorbidities were assessed once before diagnosis and changes in comorbidity status after cancer treatment could impact patient survival. Our study focused on the effect of the pre‐diagnosis comorbidity burden on mortality and its interaction with RT, aiming to provide evidence for treatment decision‐making. However, we acknowledge that self‐reported comorbidities up to 24 months before cancer diagnosis may underrepresent comorbidity burden at the time of diagnosis, as new comorbid conditions could arise between survey completion and cancer diagnosis. Fourth, there may be insufficient statistical power in the subgroup analysis due to (1) the small sample size in the groups of moderate and high comorbidity burden and (2) lower cancer‐specific death rates in those classes. Lastly, the generalizability of the study results is limited, as the participants in MHOS surveys are restricted to the Medicare managed care beneficiaries,^60^ which is a part of the Medicare program.

There are several strengths of the study to be noted. First, our study utilizing a national sample of older women would improve the generalizability of the evidence regarding the effects of RT omission on survival outcomes in older women with varying degrees of comorbidity burden. Second, follow‐up periods were relatively long compared to other studies. Considering a 15‐year survival rate of 80% with LRBC, studies with a short‐term follow‐up period (5‐year and 10‐year) can miss significant numbers of events. Therefore, our study expanded the follow‐up period beyond 10 years. Third, we used propensity score‐based weighting (i.e., IPTW) and selected women with LRBC only to provide a more valid causal inference from this observational epidemiologic study, otherwise prone to confounding and selection bias. Fourth, we showed the potential use of patient‐reported outcomes data in risk stratification for RT treatment decision‐making. This could improve the identification of older women who can omit RT.

In summary, our current patient‐reported outcomes comparative effectiveness study provides empirical evidence supporting RT omission among older women with LRBC and moderate or high comorbidity burden. For older women with low comorbidity burden, further evaluation of long‐term outcomes is warranted in prospective randomized studies. Collecting and utilizing patient‐reported outcomes data can be an effective tool for risk stratification for adjuvant RT after BCS. It will improve patient satisfaction as they are involved in treatment decision‐making.

## AUTHOR CONTRIBUTIONS

**Eunkyung Lee:** Conceptualization (equal); data curation (equal); formal analysis (equal); funding acquisition (equal); investigation (equal); methodology (equal); project administration (equal); validation (equal); writing – original draft (equal); writing – review and editing (equal). **Robert B. Hines:** Investigation (equal); methodology (equal); validation (equal); writing – review and editing (equal). **Jianbin Zhu:** Data curation (equal); formal analysis (equal); methodology (equal); software (equal); visualization (equal); writing – review and editing (equal). **Michael Rovito:** Investigation (equal); writing – original draft (equal); writing – review and editing (equal). **Kavita V. Dharmarajan:** Investigation (equal); writing – review and editing (equal). **Madhu Mazumdar:** Methodology (equal); writing – review and editing (equal).

## FUNDING INFORMATION

This study was supported by the University of Central Florida (UCF) Office of Research (EL).

## CONFLICT OF INTEREST STATEMENT

The authors declare no conflicts of interest.

## ETHICS STATEMENT

The University of Central Florida Institutional Review Board approved this study (IRB number: SBE‐18‐14238).

## Supporting information


Figure S1.

Figure S2.

Data S1.

## Data Availability

The data used in this study are available in the National Cancer Institute's repository, https://healthcaredelivery.cancer.gov/seer‐mhos/. Investigators interested in using SEER‐MHOS data must submit a research protocol as part of their data request and obtain permission.
