# Supplementary material for: Association between adjuvant radiation treatment and breast cancer‐specific mortality among older women with comorbidity burden: A comparative effectiveness analysis of SEER‐MHOS
Source: Cancer Med. 2023 Sep 14;12(18):18729–44. doi: 10.1002/cam4.6493 (PMC10557861; doi:10.1002/cam4.6493)
Supplement: Supplementary file 1 — Figure S1. [file CAM4-12-18729-s003.docx]

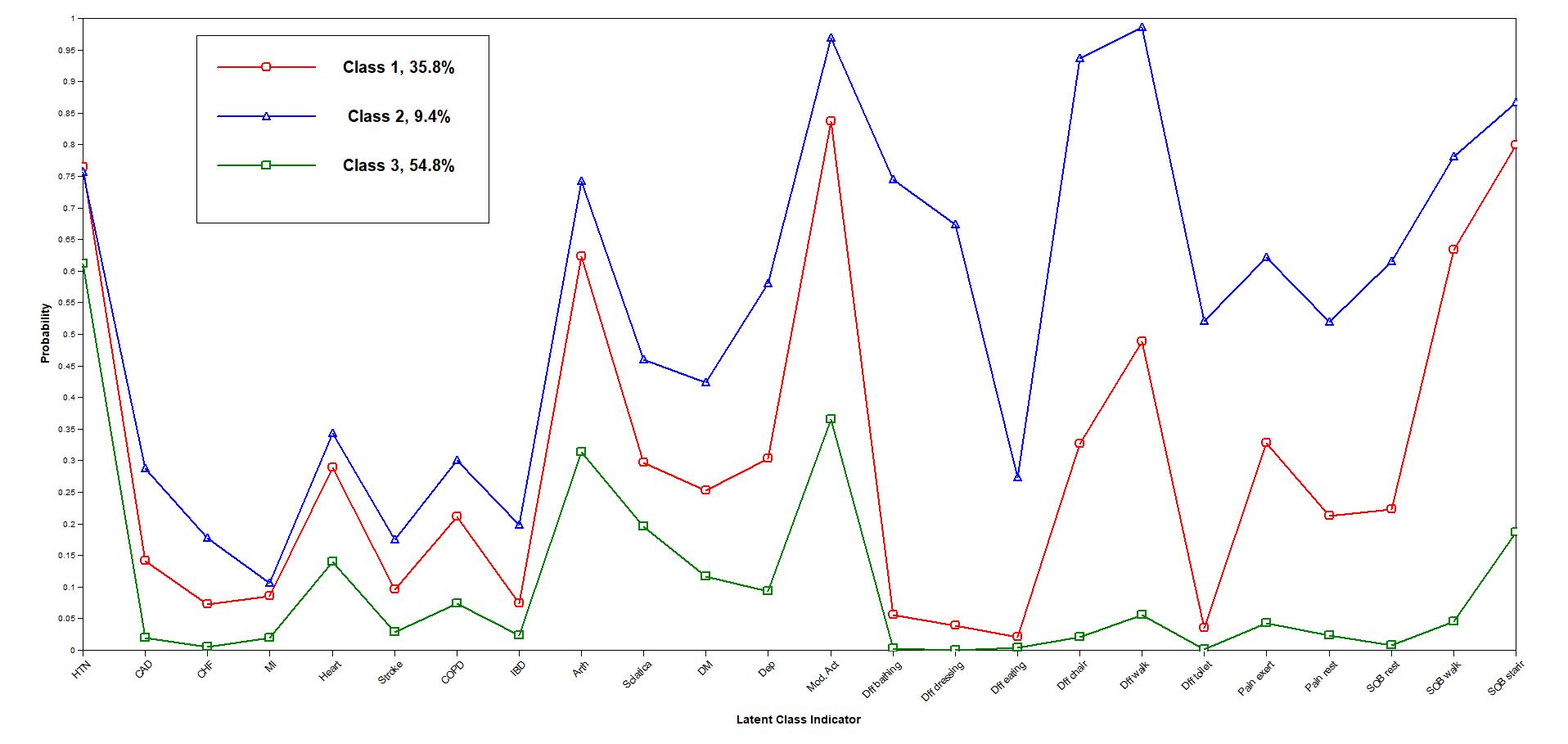


**Supplementary Figure 1**. T**he prevalence of three latent classes and the item response probability conditional on their class membership based on the estimated model.** Class 1 = “Moderate” comorbidity burden group; Class 2 = "High” group; Class 3 = "Low” group.
