# Supplementary figures and images for: Association between adjuvant radiation treatment and breast cancer‐specific mortality among older women with comorbidity burden: A comparative effectiveness analysis of SEER‐MHOS
Source: Cancer Med. 2023 Sep 14;12(18):18729–44. doi: 10.1002/cam4.6493 (PMC10557861; doi:10.1002/cam4.6493)

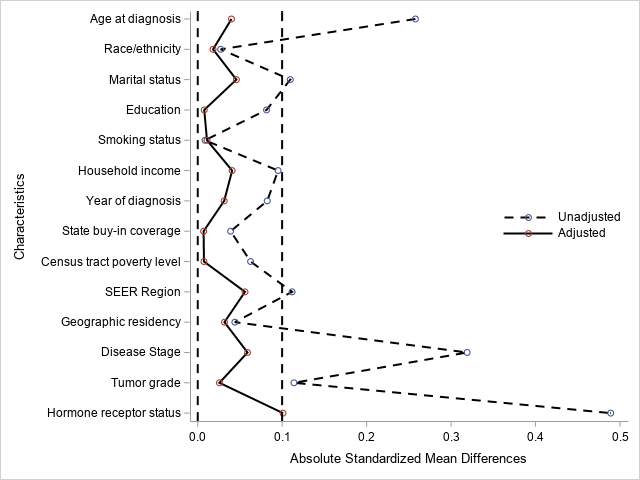

Supplement: Supplementary file 2 — Figure S2. [file CAM4-12-18729-s002.png]
