# Supplementary material for: Association between adjuvant radiation treatment and breast cancer‐specific mortality among older women with comorbidity burden: A comparative effectiveness analysis of SEER‐MHOS
Source: Cancer Med. 2023 Sep 14;12(18):18729–44. doi: 10.1002/cam4.6493 (PMC10557861; doi:10.1002/cam4.6493)
Supplement: Supplementary file 3 — Data S1. [file CAM4-12-18729-s001.docx]

**Supplementary Table 1 IPTW-adjusted subdistribution hazard ratios for the association between treatment and all-cause mortality, overall and by comorbidity burden class among older women with low-risk breast cancer, SEER-MHOS**

|  | **5-year outcomes** | | | | **10-year outcomes** | | | **20-year outcomes** | | | |
| --- | --- | --- | --- | --- | --- | --- | --- | --- | --- | --- | --- |
|  | **HR** | | **95% CI** | ***P* Value** | **HR** | **95% CI** | ***P* Value** | | **HR** | **95% CI** | ***P* Value** |
| **All (n=1105)** | |  |  |  |  |  |  | |  |  |  |
| BCS + RT | | Ref |  |  | Ref |  |  | | Ref |  |  |
| BCS Only | | 1.49 | (1.14, 1.95) | 0.004 | 1.41 | (1.17, 1.71) | <.001 | | 1.64 | (1.39, 1.92) | <.0001 |
| **Low comorbidity burden (n= 614)** | | |  |  |  |  |  | |  |  |  |
| BCS + RT | | Ref |  |  | Ref |  |  | | Ref |  |  |
| BCS Only | | 1.15 | (0.73, 1.83) | 0.548 | 1.27 | (0.93, 1.73) | 0.131 | | 1.66 | (1.31, 2.11) | <.0001 |
| **Moderate comorbidity burden (n=392)** | | | |  |  |  |  | |  |  |  |
| BCS + RT | | Ref |  |  | Ref |  |  | | Ref |  |  |
| BCS Only | | 1.62 | (1.09, 2.42) | 0.017 | 1.41 | (1.06, 1.87) | 0.017 | | 1.47 | (1.14, 1.89) | 0.003 |
| **High comorbidity burden** | |  |  |  |  |  |  | |  |  |  |
| BCS + RT | | Ref |  |  | Ref |  |  | | Ref |  |  |
| BCS Only | | 1.86 | (0.99, 3.47) | 0.053 | 1.87 | (1.16, 3.01) | <.001 | | 2.33 | (1.48, 3.65) | <.001 |
| Abbreviations: IPTW, inverse probability of treatment weighing; BCS, breast-conserving surgery; RT, radiation therapy; HR, hazard ratio; CI, confidence interval; NE, not estimable because no death was observed in BCS Only group.  IPTW model was built with age at diagnosis, race/ethnicity, marital status, year of diagnosis, insurance (state buy in), household income, census tract poverty level, marital status, education, urban/rural designation, SEER region, disease stage, tumor grade, PR-status.  Significant findings are in bold. | | | | | | | | | | | |

**Supplementary Table 2 IPTW-adjusted subdistribution hazard ratios for the association between treatment and non-cancer-specific mortality, overall and by comorbidity burden class among older women with low-risk breast cancer, SEER-MHOS**

|  | **5-year outcomes** | | | | **10-year outcomes** | | | **20-year outcomes** | | | |
| --- | --- | --- | --- | --- | --- | --- | --- | --- | --- | --- | --- |
|  | **HR** | | **95% CI** | ***P* Value** | **HR** | **95% CI** | ***P* Value** | | **HR** | **95% CI** | ***P* Value** |
| **All (n=1105)** | |  |  |  |  |  |  | |  |  |  |
| BCS + RT | | Ref |  |  | Ref |  |  | | Ref |  |  |
| BCS Only | | 1.84 | (1.35, 2.51) | <.001 | 1.56 | (1.27, 1.93) | <.001 | | 1.69 | (1.42, 2.01) | <.0001 |
| **Low comorbidity burden (n= 614)** | | |  |  |  |  |  | |  |  |  |
| BCS + RT | | Ref |  |  | Ref |  |  | | Ref |  |  |
| BCS Only | | 1.22 | (0.71, 2.01) | 0.467 | 1.24 | (0.87, 1.77) | 0.236 | | 1.52 | (1.16, 1.99) | 0.002 |
| **Moderate comorbidity burden (n=392)** | | | |  |  |  |  | |  |  |  |
| BCS + RT | | Ref |  |  | Ref |  |  | | Ref |  |  |
| BCS Only | | 2.15 | (1.35, 3.42) | 0.001 | 1.64 | (1.21, 2.23) | 0.002 | | 1.65 | (1.27, 2.16) | <.001 |
| **High comorbidity burden** | |  |  |  |  |  |  | |  |  |  |
| BCS + RT | | Ref |  |  | Ref |  |  | | Ref |  |  |
| BCS Only | | 2.26 | (1.18, 4.36) | 0.015 | 2.24 | (1.37, 3.66) | 0.001 | | 2.77 | (1.74, 4.39) | <.0001 |
| Abbreviations: IPTW, inverse probability of treatment weighing; BCS, breast-conserving surgery; RT, radiation therapy; HR, hazard ratio; CI, confidence interval; NE, not estimable because no death was observed in BCS Only group.  IPTW model was built with age at diagnosis, race/ethnicity, marital status, year of diagnosis, insurance (state buy in), household income, census tract poverty level, marital status, education, urban/rural designation, SEER region, disease stage, tumor grade, PR-status.  Significant findings are in bold. | | | | | | | | | | | |
